# Maintaining Sense of Purpose in Midlife Predicts Better Physical Health

**DOI:** 10.1093/geroni/igab046.821

**Published:** 2021-12-17

**Authors:** Emily Willroth, Daniel Mroczek

**Affiliations:** Northwestern University, Chicago, Illinois, United States

## Abstract

Having a sense of purpose in life is fundamental to psychological and physical well-being. Despite the myriad benefits of purpose, it may be difficult to hold onto purpose as people age and experience fewer future-oriented goals. The present research used reliable change indices to estimate change in sense of purpose during midlife in three diverse samples. On average, sense of purpose declined slightly with age in all three samples. Next, we used linear regression to examine associations between sense of purpose levels and sense of purpose change and later self-reported physical health outcomes. Consistent with our preregistered hypotheses, higher sense of purpose predicted better health in the two larger samples and more positive sense of purpose trajectories better health in all three samples. Together, these findings suggest that both having a sense of purpose and holding onto it may be important for physical health in middle to older adulthood.

